# In Memoriam

**Published:** 1884-06

**Authors:** 


### In Memoriam.

Dr. John B. Kidd, D.D.S., deceased, of Lexington, Ky., was born in Woodford Co., Ky., August 9th, 1853; was about thirty years of age at his death. His educational advantages had not been extensive ; he had, however, attended school in Versailles and Lexington, after which he was for some time engaged in the drug business. His mind inclining to reach out in another direction, at the age of about twenty years, he took up the study of dentistry ; first under the instruction of Dr. Floorer, of Lexington, Ky., and afterwards under Dr. O. B. Doyle, of Louisville, Ky. After this he established himself in practice in Versailles, in which he continued for three years. Not being satisfied with his professional attainments he entered the Ohio College of Dental Surgery, from which he graduated March 2d, 1876, with marked honor, soon after which he entered on the successful practice of his profession in Lexington.

Dr. Kidd was a man of warm and generous impulses, one upon whom the utmost reliance could be placed ; free, frank, outspoken, and honest in all his converse and dealings with his fellow-men. His devotion to his family, and particularly to his sisters and widowed mother, was deep and earnest.

He was fast gaining for himself friends among his professional brethren, and doubtless would ere long have made for himself distinction. Though a young man of healthy appearance he contended all his life, more or less, with a disease which at the last stopped the life current at its fountain, his heart being the seat of his disease. He was stricken down in the midst of professional labor, dying with a patient in hand.

He was an honored member of the Lexington Guards, and by them his remains were borne to their last resting place. He will ever be kindly remembered by all who knew him.

